# *Ex vivo *production of red blood cells from human cord blood

**DOI:** 10.1186/1753-6561-9-S9-P67

**Published:** 2015-12-14

**Authors:** Marta Caminal, Juan P Labrozzi, Irene Oliver-Vila, Martí Alzaga-Gragera, Silvia Marín-Gallén, Arnau Pla, Joan García, Joaquim Vives

**Affiliations:** 1 Divisió de Teràpies Avançades/XCELIA, Banc de Sang i Teixits, Barcelona, Spain

## Background

Transfusion of red blood cells (RBC) is the only clinically effective therapeutic approach for treating oxygen transport deficits (i.e. blood loss in surgical interventions and anaemia). However potential shortage of transfusable RBC has been predicted for the near future as a result of an imbalance between supply and demand due to aging population, an increase in the transmission of infectious diseases, limited compatibility of stored stocks, and the requirement for rare blood groups [1,2]. This situation has a direct impact in Public Health and it has consequently spurred the development of novel technologies for the generation of blood substitutes. The candidate products for human use should be safe, display adequate profiles for the uptake, transport and delivery of oxygen, a prolonged half-life in the bloodstream, stability at room temperature that would facilitate cost-effective storage, and they must be obtained under Good Manufacturing Practice (GMP) quality standards. In vitro production of RBCs [3-5] from hematopoietic stem/progenitor cells (HSC) [6,7], embryonic stem (ES) cells [8], or induced pluripotent stem (iPS) cells [9] under controlled culture conditions offers a potential solution to overcome this medical and social issue. However mass production of RBC has not been attained yet. The attractiveness of developing bioprocesses for ex vivo production of RBC also resides in the fact that enucleated cells pose no risk of tumorigenicity (no matter whether RBC are derived from immortalized or pluri-/multipotent cells) and, therefore, they can be transfused without hazard into the recipient. Enucleated RBCs can be selected by size (e.g., by filtration), and impurities of nucleated cells can be eliminated by irradiation without affecting the structure and function of RBCs. Indeed, such irradiation is routinely used before transfusion in order to eliminate any remaining lymphocytes. Besides, transplantation of progenitor cells requires compatibility for major histocompatibility antigens [14], but this is not the case for enucleated RBCs, which only require the compatibility of ABO and RhD blood phenotypes.

Given the potential of stem cells to recapitulate erythropoiesis in vitro under controlled conditions in standard T-flask cultures, we transferred such methodology into stirred tank bioreactors, as the first step towards scaling the bioprocess up to the production of clinically relevant doses. Furthermore, we compared the characteristics of the cells produced in bioreactors to those obtained from traditional manual cultures.

## Materials and methods

The expansion strategy lasted 21 days and consisted of 3 stages based on the use of different media additives: 1) isolation of CD34+ cells from a fresh umbilical cord blood (CB) unit using magnetic beads (on day 0); 2) expansion of CD45+ progenitors up to day 7, using 3 IU/mL Erythropoietin (EPO, Amgen), 100 IU/mL Stem Cell Factor (SCF, Amgen), 5 ng/mL Interleukin-3 (IL-3, CellGenix), 10 µM hydrocortisone (HC, Nyoden), 330 µg/mL transferrin (Life Technologies), 10 µg/mL insulin (Life technologies), 2 mM L-glutamine (Life Technologies), 5% v/v human serum B (hSerB, Banc de Sang i Teixits)-supplemented IMDM (Lonza) medium, and 3) the subsequent maturation and enucleation of erythroblasts into erythrocytes (CD45-, CD36-, CD235+ and CD71-) using EPO/SCF/transferrin/insulin/L-glutamine/hSerB-supplemented media. Cell culture concentration was adjusted at 5x105 cells/mL every 2 days and maintained at 37ºC either in humidified 5% CO2 incubators or in stirred tank bioreactors (Applikon) at 90 rpm.

Cells were analyzed by flow cytometry at different culture times for phenotypic expression of specific surface markers using a FACSCalibur flow cytometer (Becton Dickinson). May-Grünwald-Giemsa stainings were performed to identify enucleated cells by microscopy. Erythrocytes were quantified by the Retic-Count assay (Becton Dickinson). Glucose and lactate concentrations in supernatants were determined using an YSI 2700 SELECT automated analyser (Yellow Springs Instruments), as described elsewhere [10]. Haemoglobin (Hb) content was determined by High Performance Liquid Chromatography (HPLC).

## Results and discussion

**Figure 1:**
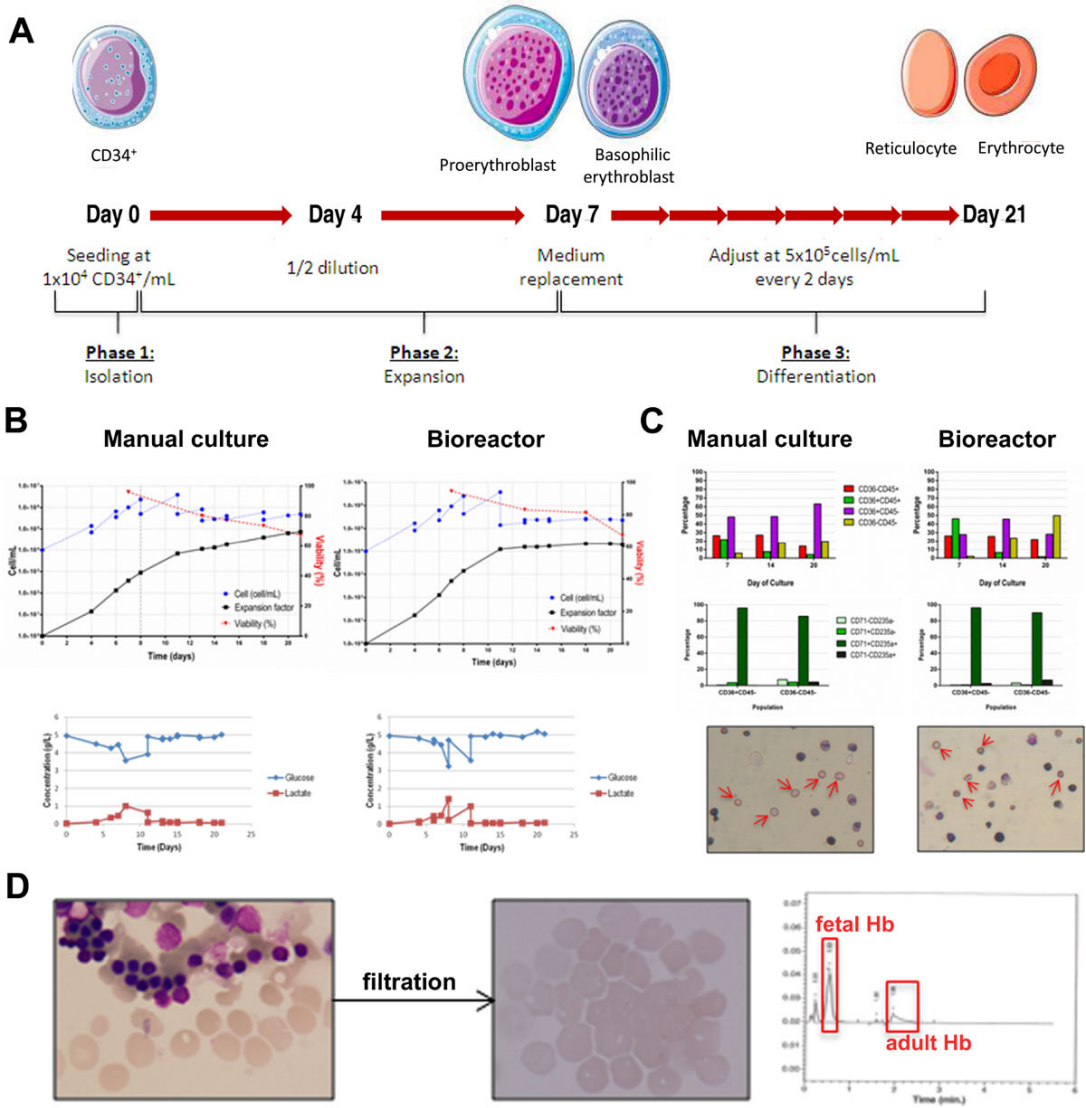
**Production strategies for the production of red blood cells in stirred tank bioreactors and T-flasks**. A. Schematic representation of the expansion strategy. B. Growth profiles and metabolic parameters in bioreactor and manual cultures. C. Characterisation of cellular products resulting from bioreactor and manual cultures. D. Characterisation of haemoglobin content (Hb) in erythrocytes produced *ex vivo*, after filtration. Part of the figure was used from ''Medical Art Gallery'' (Les Laboratoires Servier).

Considerable progress has been made in the recent years with regard to biological control of the expansion and maturation of erythroid cells with the objective of generating enucleated RBC ex vivo [11]. Giarratana et al. managed to generate large amounts of RBC in vitro from CD34+ HSC isolated from CB [12]. However, that procedure presented some limitations for its translation to the clinical setting, such as low yields and the requirement of co-culture with murine or human stromal cells. In order to overcome these issues, we developed a culture strategy that emulates erythropoieis ex vivo, also using CD34+ HSC isolated from CB as starting material, which enabled the production of enucleated erythrocytes in the absence of feeder cells. The resulting haemoglobin was composed of fetal (53 ± 12%, n = 2) and adult (26 ± 8%, n = 2) isoforms, very similar to that found in neonatal peripheral blood (72% of fetal Hb and 23% of adult Hb). The potential clinical use of this product is supported by the fact that patients suffering from inherited persistence of fetal Hb do not develop anaemia [7,13]. Both bioreactor and manual cell culture strategies displayed a similar behaviour characterised by high cellular expansion during phase 2, which was maintained during phase 3 (where maturation occurred) and high viability rates, in the range 60-90%. Glucose consumption and lactate generation showed similar profiles in both culture strategies (Figure 1).

During erythroid progenitor differentiation, CD45 expression decreased while CD36 expression appeared gradually along the culture time to eventually disappear in mature erythrocytes. The two most mature populations (CD36+CD45- and CD36-CD45-) were analysed for CD71 and CD235a expression at the end of the cultures. High levels of CD71 and CD235a are indicative of the reticulocyte phenotype, while the expression of CD71 is lost in mature erythrocytes. In terms of its clinical use, this does not pose a problem, since it has been demonstrated that reticulocytes fully mature into erythrocytes, once they are injected into the bloodstream [2,6]. Bioreactor expansion favoured culture maturation, as the percentages of most mature populations were higher.

Presence of enucleated cells were confirmed in both culture strategies by May-Grünwald-Giemsa staining. Nucleated cells were removed from the final product by filtering and the final product was composed of a pure population of biconcave in vitro-produced erythrocytes (Figure 1).

Kinetic parameters summarized in Table 1 evidenced that the enucleated cell yield per each initially seeded CD34+ cell was higher (3.2x104) in manual cultures than in bioreactors (1.34x104). However, percentages of enucleation were larger in bioreactors (67.50% in front of 44.20% in manual culture). This seems to indicate that erythroid progenitors mature faster in bioreactors, losing some of their expansion capacity, as a consequence. Concerning the use of bioreactors for erythrocytes culture a limited number of publications is available. Giarratana and collaborators reviewed some of them but it is very difficult to compare the results obtained, as a wide range of bioreactors have been tested [5].

**Table 1:** Summary of kinetic parameters.

	MANUAL CULTURE	BIOREACTOR CULTURE
**Enucleation percentage **	44.20%	67.50%
**Initial CD34^+ ^cell number**	2x10^5^	5x10^5^
**Total Expansion Factor **	7.27x10^4^	1.99x10^4^
**Theoretical number of total cells **	1.45x10^10^	9.95x10^9^
**Theoretical number of total enucleated cells**	6.41x10^9^	6.72x10^9^
**Theoretical number of enucleated cells per CD34^+^**	3.2x10^4^	1.34x10^4^

Results at day 21, from one out of 3 independent experiments. Theoretical values indicate the predicted values if media volumes were increased instead of fixed at 250 mL.

Existing cell culture technologies for RBC production are not easily transferable to large-scale settings and, moreover, cell yields using CB as starting material are far behind those needed in the clinical setting. Hence, it is not likely that the first attempts to generate massive quantities of ex vivo cultured erythrocytes will use this stem cell source. Instead, much work is currently invested on either establishing cell lines or using human embryonic stem cells (hES) and induced pluripotent stem cells (iPS), as unlimited source of HSCs [5].

## Conclusions

We demonstrated the feasibility to obtain red blood cells ex vivo from CD34+ umbilical cord blood cells and transfer the expansion strategy from manual cultures to stirred tank bioreactors. Phenotypic characterisation and enucleation rates were similar in manual and bioreactor strategies. Although cells cultured in bioreactors showed lower expansion potential, higher maturation yields were observed. Further work will focus on bioprocess optimisation and scale up to clinically significant doses.

## Acknowledgements

This work was supported by the Spanish Cell Therapy Network (TerCel, expedient number RD12/0019/0015), and the REDONTAP project was funded by the European Union's Seventh Framework Programme (grant agreement number 229328).
